# Evolving robot empathy towards humans with motor disabilities through artificial pain generation

**DOI:** 10.3934/Neuroscience.2018.1.56

**Published:** 2018-01-30

**Authors:** Muh Anshar, Mary-Anne Williams

**Affiliations:** 1 Social, Cognitive Robotics and Advanced Artificial Intelligent Research Centre, Department of Electrical Engineering, Universitas Hasanuddin UNHAS Makassar Indonesia; 2 Innovation and Enterprise Research Lab, Centre for Artificial Intelligence, University of Technology Sydney UTS Australia

**Keywords:** cognitive, empathic reaction, assistive robots, synthetic pain, joint position, shoulder motion

## Abstract

In contact assistive robots, a prolonged physical engagement between robots and humans with motor disabilities due to shoulder injuries, for instance, may at times lead humans to experience pain. In this situation, robots will require sophisticated capabilities, such as the ability to recognize human pain in advance and generate counter-responses as follow up emphatic action. Hence, it is important for robots to acquire an appropriate pain concept that allows them to develop these capabilities. This paper conceptualizes empathy generation through the realization of synthetic pain classes integrated into a robot's self-awareness framework, and the implementation of fault detection on the robot body serves as a primary source of pain activation. Projection of human shoulder motion into the robot arm motion acts as a fusion process, which is used as a medium to gather information for analyses then to generate corresponding synthetic pain and emphatic responses. An experiment is designed to mirror a human peer's shoulder motion into an observer robot. The results demonstrate that the fusion takes place accurately whenever unified internal states are achieved, allowing accurate classification of synthetic pain categories and generation of empathy responses in a timely fashion. Future works will consider a pain activation mechanism development.

## 1. Introduction

Social assistive robotics has been one of growing fields of study in human-robot interaction (HRI) that covers the utilization of robot technology to assist people through social interaction. A prolonged physical engagement between assistive robots and humans, particularly those with motor disabilities due to a shoulder injury for instance, may at times lead humans to experience pain. In this situation, robots are required to develop a more sophisticated HRI capability, namely the ability to recognize and predict human pain during an interaction, and at the same time, to adopt counter responses as empathic action when human pain experiences are predicted. Hence, it is critical for assistive robots to acquire an appropriate and relevant concept of pain that allows them to develop and generate effective empathic behaviors. This paper conceptualizes and implements the generation of empathy into two stages: (1) The realization of the concept of artificial pain through synthetic pain classes integrated into a robot self-awareness framework, and the utilization of robot body awareness to implement the fault detection as the primary source of synthetic pain activation; (2) the projection of the human shoulder motion into the robot arm motion using a fusion process that allows the gathered information to be used to generate a corresponding synthetic pain in the robot that allows it to adopt counter empathic responses.

A practical approach is designed to mirror the internal state of a human shoulder motion projected on a observer robot's shoulder. A special remark on the studies in human empathy suggests that an empathic state is obtained through perceptions towards another person's narrative. In other words, an empathic person does not directly experience what the other person is experiencing. By utilizing this concept, our approach is to have an observer robot which mirrors the internal state of the human peer by capturing the human shoulder motion through the robot's visual perception.

Our findings demonstrate that the projection of the human and the observer robot takes place accurately when they both share unified internal states. An accurate projection further allows better prediction results of the robot body behavior, accurate classification of synthetic pain categories/levels, and at the same time, appropriate generation of empathy responses in a timely fashion.

The reminder of the paper proceeds as follows: Section 2 presents an overview of related work in assistive robots highlighting self-awareness, human pain and empathy concepts. Section 3 gives a brief description of the proposed synthetic pain and empathy concepts within a self-awareness framework for robots, followed by section 4 which explains experiment stages and environmental set up. Section 5 provides evaluation and discussion. Finally, section 6 concludes the overall achievement and possible future developments.

## 2. Robot design

In this section we briefly present relevant background studies in the area of assistive robotics, self-awareness, pain and empathy concepts.

### 2.1. Assistive robotics

Assistive robotics is a growing field of study in HRI that focuses on assisting people with physical disabilities and the robots typically utilize a physical medium or physical contact when delivering assistance. As the physical contact interaction occurs, the element of embodiment plays a crucial role as the basis for a structural coupling that creates potential perturbation between robots and the environments. The design approaches commonly consider a biologically inspired approach or functionality designs which focus on the constrained operational and performance objectives [6]. Studies in assistive robotics cover a wide range of applications, such as rehabilitation robots, wheelchair robots, educational robots and manipulator arms for disable people [7]. People with physical disabilities, particularly with a motor disability, are people who suffer from conditions that restrict their abilities in moving and manipulating object tasks. This condition introduces a significant limitation in moving, controlling and coordinating the movement of body parts, such as wrist, hands, fingers or arms [2]. People with shoulder motor injury experience pain as the shoulder moves to specific positions which will evoke the sensation of pain.

### 2.2. Cognitive designs

In the theory of mind (ToM) literature, it is reported that humans have the ability to correctly attribute beliefs, goals, and perceptions towards themselves and other people [3]. A robot, with the ability to recognize human emotional, attentional, and cognitive states, can learn to develop counter reactions and modify its own behavior accordingly. This concept is central and plays a crucial role in human interactions, including in the field of assistive robotics. The mind is considered as the consciousness embodiment, where consciousness is defined as a function of consistent cognition and behavior performance [4],[5]. Proposes that embodiment is one of the features of consciousness, and our self-concept utilizes this embodiment aspect in developing our empathic robot responses.

#### 2.2.1. Self-awareness

According to [10] robots with self-awareness have the ability to behave more effectively in novel situations compared to those without it. Studies on the notion of robots being self-aware early appeared in [3],[8], where [8] develops a capability for a robot to recognize itself in the mirror. Since then, self-aware robot studies continue to grow as reported in [9],[11]–[15]. Propose a framework with a self-awareness based on the ability to focus attention on the internal state's representation. Much of literatures, however, identifies the lack of concept of “self” [1]. Propose a concept of self by deriving its definition postulated by [16] which divides the concept into two levels, subjective and objective awareness. Subjective awareness concerns the machinery level of the body and objective awareness concerns the focus of attention towards one's body, thought, actions and feelings. The authors further introduce a new framework which is capable of switching the robot awareness from subjective to objective, and vice versa.

#### 2.2.2. Empathy for the pain of others

Studies of empathy have been growing in the last decade, particularly human empathy towards pain, as reported in [17]–[23]. A common understanding is emerging that suggests a complex structure in the human brain and the nerve cells assembly play a major role in the arousal of empathy and pain. With such complexity, generating empathy and pain should be developed by considering the current state-of-the-art of robot technology. The implementation of our robot empathy for pain is inspired by the work in [24], which proposes a shared-representation model of pain empathy. This model mentions that witnessing another person in pain activates pain representations in the observer, which reflects a relative capacity to understand pain experiences in others. Hence, our empathy for pain is generated by projecting humans' body, e.g. shoulder motions which suffer from a motor injury into a robot observer's shoulder. The observer robot visually captures the shoulder motions and projects them on its own arm, while analyzing the kinds of synthetic pain to be generated.

## 3. Self-awareness framework and synthetic pain

**Figure 1.**
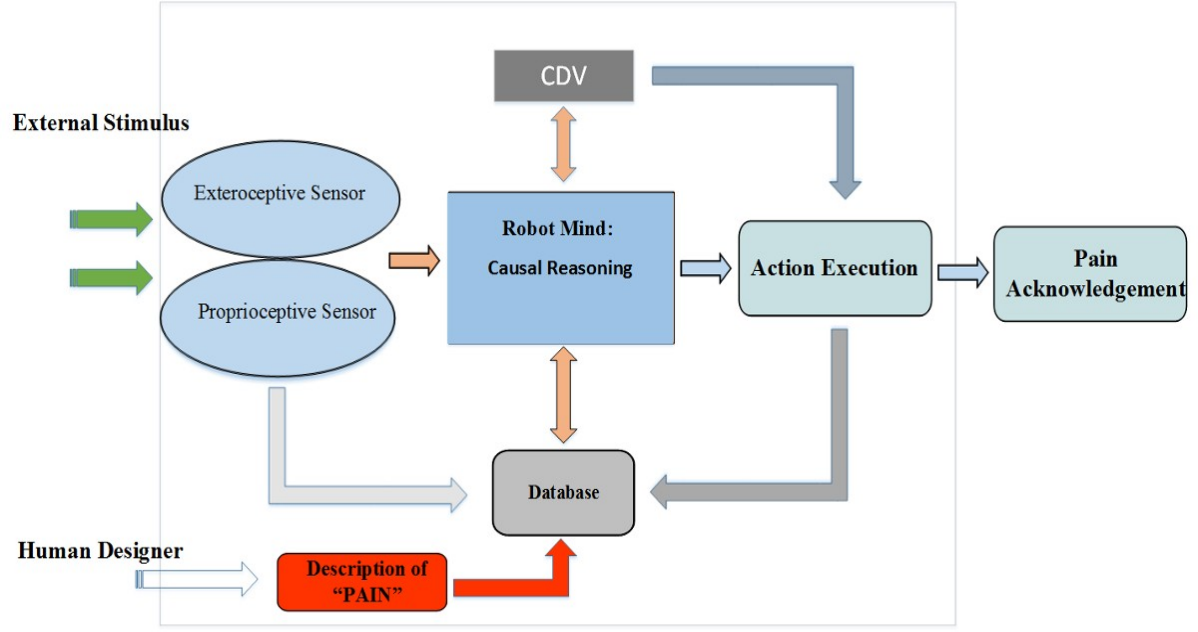
Adaptive self-awareness framework for the robot (ASAF).

This paper utilizes a self-awareness framework proposed by [1], Adaptive Self-Awareness Framework for Robot (ASAF) and the kinds of synthetic pain definitions in developing our empathic reactions. The overall design of the ASAF is shown in Figure 1 below. In this new framework, the subjective awareness refers to the element of physical parts of robots or robot embodiment, such as motors and joints; while the objective awareness specifies the metaphysical aspects, such as robot's representation of its position towards an external reference. A brief overview of the ASAF is discussed in the following subsection.

### 3.1. Adaptive Self-awareness framework for robot (ASAF)

There are three elements that play pivotal roles in the performance of the framework, which we are going to further elaborate in the following subsections.

#### 3.1.1. Consciousness direction

**Figure 2.**
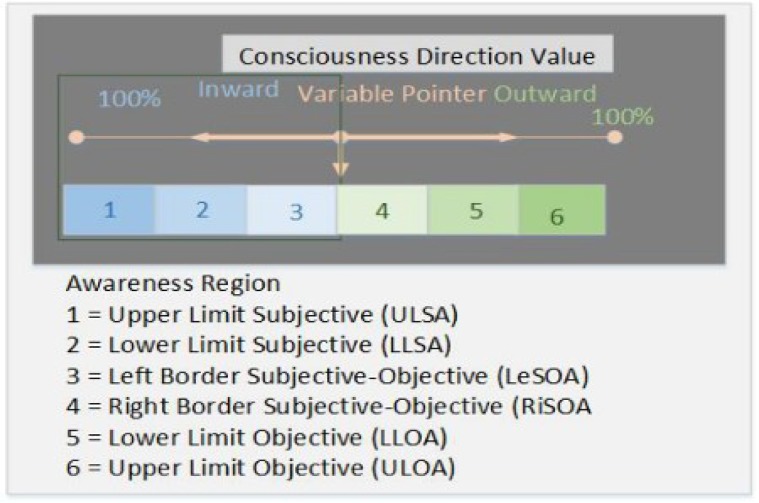
Regions of consciousness direction.

The term of consciousness in the ASAF is to signify the cognitive focus, which is the focus of attention and it should not be understood to mean human consciousness. Hence, consciousness direction is changeable between the two levels of awareness, subjective and objective awareness (see Figure 2). There are two predominant factors in directing robot consciousness: (i) The ability to focus attention on a specified physical aspect of self. (ii) The ability to foresee, and at the same time, to be aware of the consequences of predicted actions. Our approach formulates how to address these two aspects so that they can be developed and built into a robot self-awareness framework. Thus, the detection of synthetic pain can be acknowledged and responded to in an appropriate way. Robot awareness is mapped to a discrete range 1–3 for subjective and 4–6 for objective elements. Changing the value of Consciousness Direction (CDV) allows the exploration of these regions, and at the same time, changes the focus of robot attention. The robot mind governs the CDV modification and determines the conditions of exploration of robot awareness regions, either constrained or unconstrained conditions. The structure of robot awareness regions and CDV are illustrated in Figure 2 and for simplicity, we will use the abbreviations for each awareness region throughout the paper.

#### 3.1.2. Synthetic pain description

**Figure 3.**
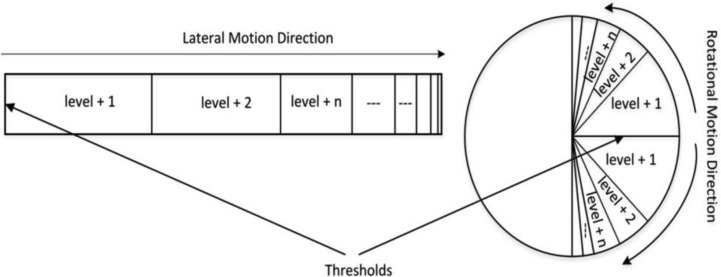
Synthetic pain generation.

In order to generate synthetic pain on the robot, we set shoulder joint restriction regions that should be avoided. People with a shoulder motor injury have specific joint regions which will evoke pain sensation whenever the shoulder moves into these areas. These joint regions are projected into the observer' shoulder joint and each restrictive joint regions constitutes a specific faulty joint value. Synthetic pain can then be generated when the robot joint moves into this region. Joint movement is monitored by the proprioceptive perception of the robot, which can subsequently be used by the robot mind to reason upon. For specific types of movement, for instance rotational movement for the shoulder joint, the pain level is determined by the current joint values with respect to the joint threshold value. This threshold value is set by the robot mind and its value is associated with the lowest fault joint value (see Figure 3).

Synthetic pain descriptions proposed by [1] have different applicable intensity level for each class (see Table 1).

**Table 1.** Synthetic pain description.

Category	Synthetic pain	Description	Definition	Intensity Level
1	Proprioceptive	1.0	Potential hardware damage, as an alert signal	“None”, “Slight”
2	Inflammatory reduction	2.1	Predicted robot hardware damage, real robot hardware damage	“None”, “Slight”
		2.2		“Moderate”, “Severe”
3	Sensory malfunction	3.1	Abnormal function of internal sensors, damage internal sensors	“None”, “Slight”
		3.2		“Moderate”, “Severe”

#### 3.1.3. Robot mind

The robot mind in the framework utilises causal reasoning to draw conclusions from its perceptions which integrates the cause and effect relationship. This allows the framework to adapt to the world by predicting the robot's own future states through reasoning about the perceived/detected facts. Sequential pattern prediction is used to capture the behavior of the observed facts and then use them to predict the possible future conditions. The framework decision making utilizes covariance information obtained from sequence data so as to facilitate the causal reasoning process. The robot mind analyses the relationship amongst data covariance by making predictions of sequence data patterns obtained from robot's proprioceptive sensor (joint position sensor). The prediction process only takes place after several sequences of data so as to reduce biased analyses. There are two conditions of the state of the robot mind:

1. Constrained which represents a state where the CDV value is restricted to subjective awareness, region 1 (upper level). This state typically will force the robot to stop any physical activity on the hardware level.

2. Unconstrained which refers to a state where the CDV value is free to explore all the regions of the robot awareness (region 1 to region 6).

### 3.2. Empathic actions generation and execution

There are two stages that occur in the robot mind in the specific example of human shoulder pain: (1) The realization of the concept of artificial pain through synthetic pain classes integrated into the ASAF framework, and the utilization of robot body awareness to implement the fault detection as the primary source of synthetic pain activation; (2) the projection of the human shoulder motion into the robot arm motion using a projection process that allows the obtained information to be used to generate a corresponding synthetic pain in the robot and counter empathic responses. Stage 2 occurs at the first place as the observer robot projects the human shoulder motion into the robot's arm and simultaneously stage 1 occurs. Joint data is captured from the robot proprioceptive sensor attached to the shoulder joint and arranged into a sequence of data for pattern analyses. The robot mind analyses the relationship among data covariance by making predictions of sequence data patterns. The prediction process only takes place after several sequences of data so as to reduce biased analyses. Any decisions made from previous sequence predictions are reassessed with the current state, and the results are either kept as history for future prediction or amendment actions take place before placement proceeds. This cycle repeats only if current and predicted data are not classified in any of the restricted regions that refer to the painful joint settings. Once the reasoning indicates that the joint motions are heading towards or falls into these restricted joint regions, the robot mind will perform three consecutive actions:

1. Setting the robot awareness into constrained condition.

2. Modifying the CDV to shift robot's focus of attention to the subjective element, which is the robot shoulder.

3. Providing empathic response actions, such as alerting the human peer through verbal expressions and approaching the human peer for further assistance.

At any initial state, the robot mind specifies the awareness to a randomized style, which means that the attention may focus on one of among the sixth regions by randomly selecting the CDV. Once a selection is made, the robot mind is set to an unconstrained condition, allowing the robot to start visualizing and projecting the human shoulder motions. While the awareness is on the selected region and projection takes place, the robot mind at the same time monitors its proprioceptive sensor, joint arm sensors which physically project the human shoulder positions. The change of joint sensor readings produces the change in the pattern, and this situation is captured and used as the element of reasoning. As the joint moves, the robot's internal states are subjects to changes and the empathic action executions transforms the results into primitive actions for further execution.

## 4. Experimental design

**Figure 4.**
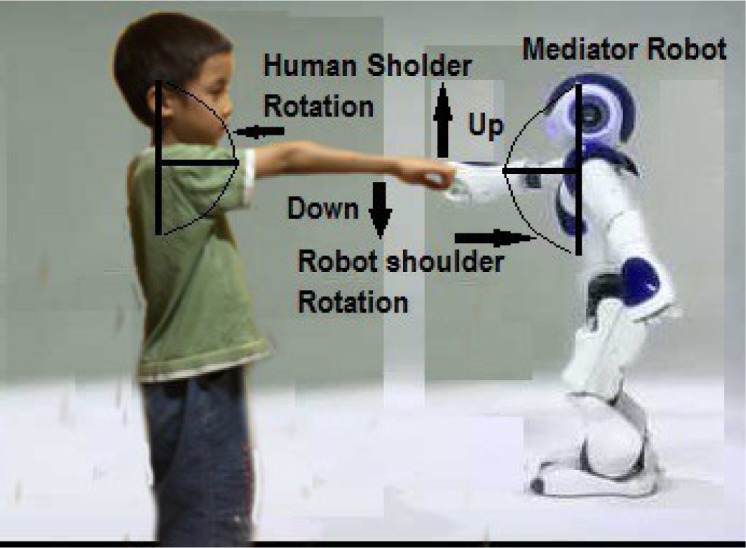
Human and mediator robot shoulder rotation mapping.

The pilot experiment considers only a two-direction up and down rotational motion of the human's right shoulder. A set of pre-defined joint values which constitute the restricted joint values are “manually” specified on the robot. These joint values are associated with the painful regions of the human's shoulder which are supposed to be avoided. The experiment involves two NAO humanoid robots and a human peer. The scenario of experiment is a human peer and one NAO humanoid robot (mediator robot) are working together in a collaborative task, e.g. a hand pushing task, while the other NAO robot acts as an observer robot. The length of the rotation movement of the human shoulder follows the length of shoulder rotation of the mediator robot. We attach a red mark on the back side of the mediator robot, which will be recognized by the observer robot via its camera sensor.^1^ During the experiment, the human's hand coincides with the mediator's hand, allowing both hands to move in parallel. Each human's shoulder rotation corresponds to the value of the mediator' shoulder joint position sensor, which is recorded in the mediator's Robot Mind (see Figure 4).

**Figure 5.**
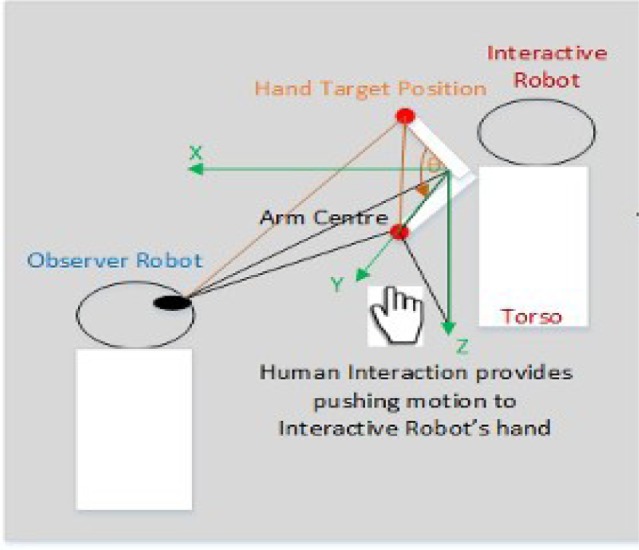
Experimental setup.

**Figure 6.**
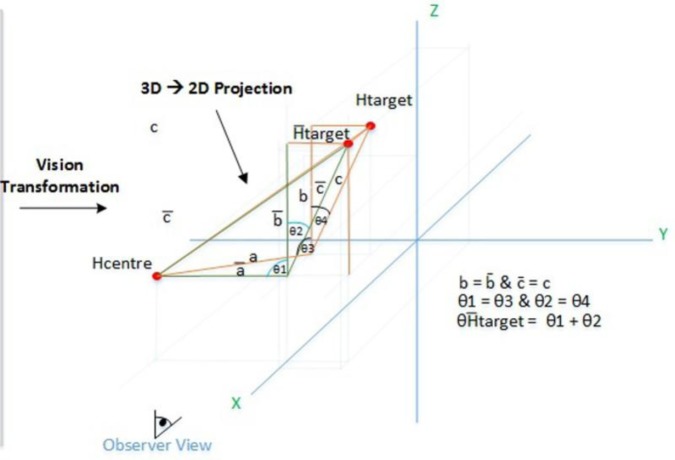
Geometric transformation.

This recorded data is utilized for merely a comparison purpose in the data analyses which is presented in this section. The observer converts the visual representation using the geometric transformation (see Figure 5 and Figure 6).

**Figure 7.**
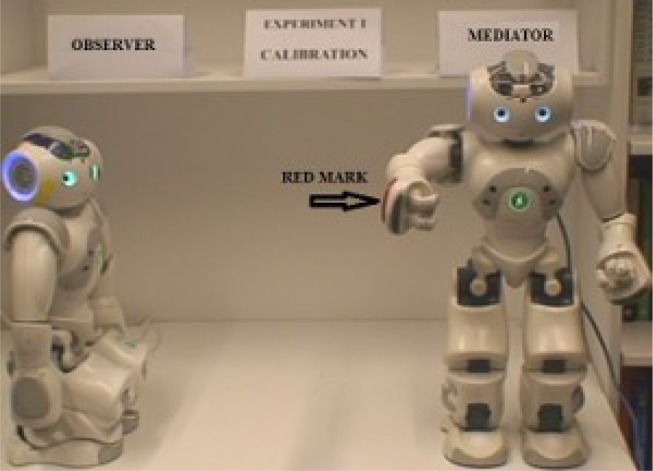
Initial pose of robot experiments.

**Figure 8.**
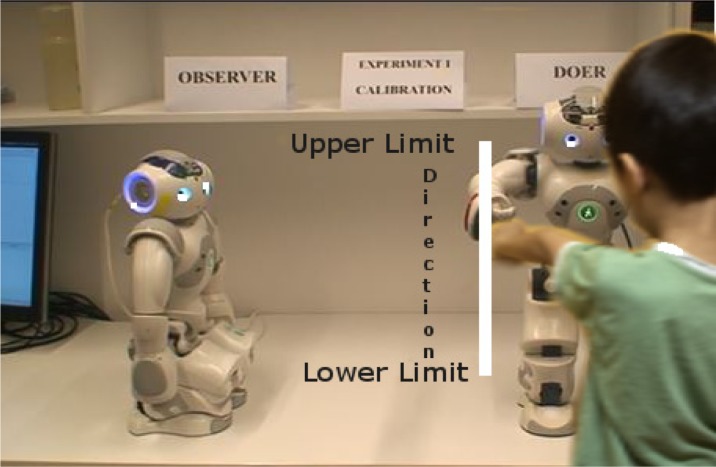
Experimental setting during interaction.

The experiment is divided into two stages. Stage 1 serves as an initiation or calibration stage. Stage 2 is the projection stage which consists of self-reflection without awareness and empathic experiments. The poses of the two robots are shown in Figure 7 and Figure 8 shows the pose of human during interaction.

During self-reflection experiment, the ASAF framework is deactivated in the observer. Instead, it only activates detection of faulty hardware region without anticipations' follow-up. The rest of experiment involves the implementation of a full functional ASAF framework.

The kinds of synthetic pain and awareness region to be modelled during this experiment are shown in Table 2 and Table 3 respectively (See Figure 2 in Subsection 3.1 for details of each region).

**Table 2.** Consciousness region.

Consciouness Region		Robot Action	During Visitation
		Unconstrained	Constrained
Subjective	ULSA	Low Stiffness on Arm Joint	Increased Stiffness and Alert human peer
	LLSA	-	-
Subjective-Objective	LeSOA	-	-
	RiSOA	-	-
Objective	LLOA	-	-
	ULOA	-	-

**Table 3.** Synthetic pain experiment.

Synthetic Pain		Descriptions	Intensity Level
Proprioceptive	1.0	Modelled: “None”	Modelled: “Slight”
Inflammatory Reduction	2.1	Modelled: “None”	-
	2.2	-	-
Sensory Malfunctions	3.1	-	-
	3.2	-	-

## 5. Results and discussions

Three kinds of data: (1) Awareness region; (2) faulty joint region; (3) arm coordinate centre reference data, are collected in the initiation stage.

### 5.1. Experiment results

The awareness and faulty joint regions are defined before the experiment takes place, and stage 1 provides the shoulder shared reference point between the mediator and the human peer. These values are the same for the stage 2 of the experiment (see Table 4).

**Table 4.** Regions and data references.

Element	Region/Ordinate	Values
awareness region	ULSA	1
	LLSA	50
	LeSOA	51
	RiSOA	100
	LLOA	101
	ULOA	150
faulty joint region	1 Upper High-UH	−2.08313
	2 Upper Medium-UM	−1.58313
	3 Upper Low-UL	−1.38313
	4 Lower Low-LL	1.385669
	5 Lower Medium-LM	1.585669
	6 Lower High-LH	2.085669
shoulder mediator human shared reference	X	0.408
	Y	−0.155
	Y	0.186
	Time	154.98

**Table 5.** Self-Reflection direction.

Direction							
Up				Down			
Observer	Human	Interval	Status	Observer	Human	Interval	Status
	Shoulder				Shoulder		
−0.015	−0.01845	0	-	1.173	0.01845	0	-
−0.015	−0.01845	340	-	1.349	0.10742	762	-
−0.015	−0.01845	664	-	1.614	1.00941	1150	-
−0.422	−0.06439	991	-	-	-	-	-
−0.94	−0.43408	1469	-	-	-	-	-
−1.506	−1.21489	1992	-	-	-	-	-
−1.941	−1.63213	2530	-	-	-	-	-
	−1.63674	3064	-	-	-	-	-

The stage 2 experiment generates two sets of data, self-reflection data (Table 5) and empathic data (Table 6).

Table 7 provides additional data obtained from the empathy experiment. This data shows the changes in the embodiment elements that affect the internal state of the observer.

**Table 6.** Empathic experiment function.

	Observer			Human Shoulder		
	Interval	Data	Prediction	Interval	Data	Status
Up	12	1.214		891	0.01845	
	13	−0.291		2185	−0.21625	
	14	−0.777		2904	−0.55527	
	15	−1.165	−1.553	4849	−1.97268	
	16		−1.941	5522	−2.08567	
	17		−2.329	-	-	
	18		−2.717	-	-	
Down	12	0.242		509	0.01692	
	13	1.186		1240	0.35133	
	14	1.805		1890	1.33922	
	15	2.105	2.405	2537	1.83317	
	16		2.705	3186	2.08567	Not Exist
	17		3.005	-		
	18		3.305	-		

**Table 7.** Internal state.

Motion	CDV	Awareness		Status	Faulty Joint Region		Internal
		Region	Early Type		Real	Prediction	State
Down	131	6	ULOA		4		1
	33	2	LLSA		4		2
	17	1	ULSA	Unconstrained	6		3
	80	4	RiSOA		6 (2.105)	6 (2.405)	4
	110 to 3	5 to 1	LLOA	Constrained	6 (2.405)		5
Up	2	1	ULSA		4		6
	62	3	LeSOA		3		7
	116	5	LLOA	Unconstrained	3		8
	6	1	ULSA		3(−1.165)	2 (−1.553)	9
	126 to 6	6 to 1	ULSA	Constrained	1(−1.941)		10

### 5.2. Analysis and discussion

**Figure 9.**
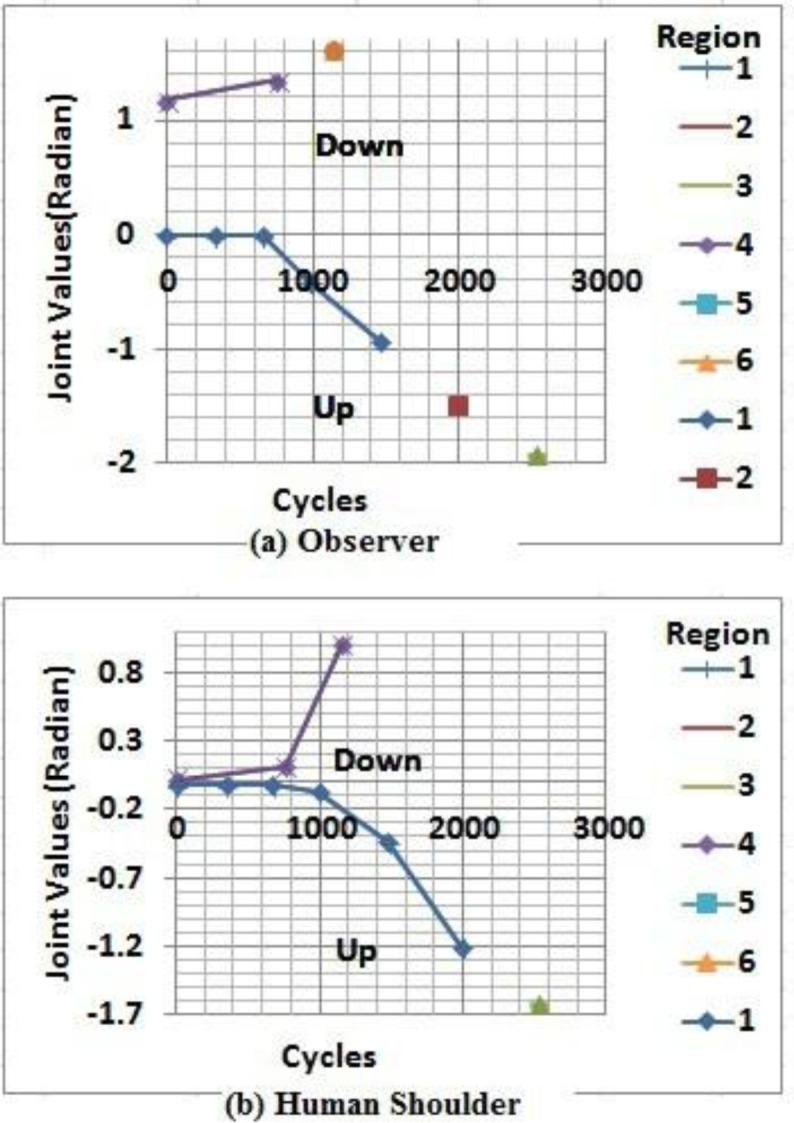
Data mapping onto hardware faulty region.

**Figure 10.**
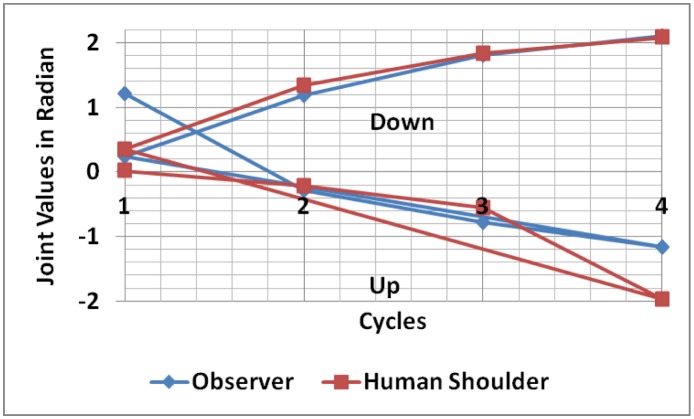
Empathy data conversion comparison.

**Figure 11.**
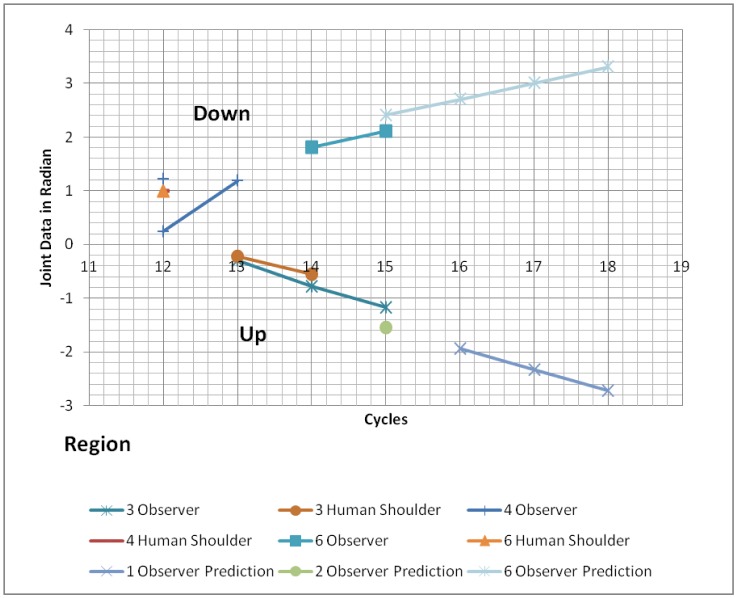
Empathy data mapping onto faulty region.

During the self-reflection experiment, the minimum sampling time required to capture incoming data, as shown in Figure 9(a) and Figure 9(b), is every 340 cycle of data. For the observer during upward motion direction, the first five sequences of data falls into Region 1 followed by Region 2 (data = −1.506, sequence = 1992), then Region 3 (data = −1.941, sequence = 2530). When data equals −1.506, the observer starts to experience Category 1 synthetic pain, which produces an alert signal (projecting the human shoulder would experience the same condition). However, the observer still captures incoming data which shifts to Region 3, causing the synthetic pain category to increase to Category 2 with the detail of 2.1. It can be seen that the sampling time tends to increase, from 327 to 478, 523 and finally 538 cycles of data as the joint falls into synthetic pain region. At the same time, during upward motion direction, the first six data sequence of human shoulder positions are classified into Region 1 (synthetic pain Category 1) and the final sequence falls into Region 3. This increasing sampling time occurs as more computation time is required by the observer to critically analyze and predict the plausible future internal state of the human shoulder position. During this upward motion direction, particularly in sequence 1992, the observer produces one false alarm, which generates Region 2 classification, while the human shoulder position is still in Region 1. However, both confirm that at sequence 2530, the internal state converges into Region 3. During the downward trend, the experiment lasts a short time, and the observer, unfortunately, misclassifies the internal state of the human shoulder position at sequence 1150. In this sequence, the observer internal state classifies Region 6 while the human shoulder position is Region 4. However, both of them share the same classification results, Region 4 which occurs at sequence 0 and 762. For the empathy experiment, the vision data comparison shown in Figure 10 and the empathic response experiment is shown Figure 11.

It can be seen from Figure 10, regeneration of the human shoulder data is similar, particularly during the downward trend, with a relatively low Δerror = 0.07751. A slight variant occurs during the upward trend with a considerable Δerror about 0.57492. These data discrepancies influence the reasoning process as any small fraction of data affects considerably prediction results.

Figure 11 shows, during the upward experiment, that the observer prediction starts at interval data 15 with the observer data is Region 3 (data = −1.165). However, prediction data at this time is Region 2 (data = −1.553) which still misclassifies the real data on the human shoulder (Region 1, data = −1.973, generated synthetic pain is Category 1.0). However, in the interval data 16, both observer prediction and human shoulder converge into Region 1 (observer data = −1.973, prediction data = −1.941 and human shoulder data = −2.086). This situation accurately is matched with the real data on the human shoulder position, and finally ends at Region 1 with synthetic pain Category 2, detail 2.1. The behavior of internal states of both robots during the downward experiment indicates a similar pattern. The observer prediction and real data nearly converge to the same pain region classification, Region 6, at interval data 15 with the observer data prediction is 2.405, observer data itself equals 2.105 and human shoulder data = 1.83317. Thus, it generates the kind of synthetic pain Category 2, details 2.1 forcing the robot to provide an alert signal. When the next interval data 16, the robot has already prepare an empathy response as the prediction data already shows an increase pattern of the observer data. The changes in the observer internal states show in Table 8.

**Table 8.** State of awareness, synthetic pain and empathy response generations.

Internal State	Awareness	Synthetic Pain		Empathy Response	
	Final Type	Categories	Intensity	Direct	Follow-Up
1	ULOA		-	-	-
2	LLSA		-	-	-
3	ULSA	No Pain	-	-	-
4	RiSOA		-	-	-
5	ULSA	1:0 Proprioceptive	“Slight”	painfully restricting	Approach the Scene
6	ULSA		-	-	-
7	LeSOA		-	-	-
8	LLOA	No Pain	-	-	-
9	ULSA		-	-	-
10	ULSA	1:0 Proprioceptive	“Slight”	right arm is mid	Alert Approach the Scene

When the observer internal state is State 3, the awareness state switches to high priority subjective awareness, and the robot mind state is unconstrained. This state demonstrates that the robot mind may focus its attention on its embodiment aspects regardless of any synthetic pain being identified. The final awareness states for both, upward and downward experiments converge to high priority subjective awareness, forcing the robot attention to focus on the arm joint of the observer, and at the same time projecting the same situation on the human shoulder. A major difference occurs in the empathy response of the observer, in which during the upward experiment, direct impact of the projection forces the arm joint stiffness in a painful state as the observer prediction produces a false alarm at early stage of the experiment. While in the downward experiment, the observer accurately projects the internal state of the human shoulder, resulting in an accurate early alert information of an incoming synthetic pain experience on the human shoulder. This situation gives an advantage for the observer to provide an early direct empathy response by setting the arm joint stiffness to a medium level, and at the same time, to allow an adequate time for approaching the human peer and offer an assistance.

## Conclusion

This paper implements robot empathy for synthetic pain by modelling robot self-awareness on subjective and objective elements. The embodiment of the consciousness feature through robot body part motions is integrated into the Adaptive Robot Self-Awareness Framework (ASAF) of a robot. We demonstrate that a robot can use ASAF, to build a projection of the internal state of agents apart from itself, and later on, to develop appropriate empathy responses. The projections allow the robot to simulate and experience the other robot's internal state, develop an accurate pain description, and generate appropriate empathy responses. Data transformation captured through robot vision determines the quality of projection process. The causal reasoning through sequential pattern prediction enables the robot's decision making to embrace the past, the current and the future considerations. This ability allows the robot to build expectations of the other agent's internal state. The observer's focus of attention switches as the reasoning process predicts the synthetic pain level that the human shoulder is experiencing.

Overall, the projection takes place accurately when both robots share a unified internal state. As the robot mind of the observer predicts that the element of body moves into the faulty joint region, the computation time increases and as a result, introduces data analyses discrepancies through a false alarm generation. This false projection occurs due three main causes: (i) Limited data to be used in sequence data prediction process decreases the quality of reasoning; (ii) the hardware discrepancies of the arm joint motor motion areas; (iii) there is a variable speed in hand movement of the human peer. The experiment also shows that robot awareness may revisit any of its consciousness regions under the unconstrained condition unless the robot mind switches to constrained condition. Utilization of the ASAF demonstrates a foreseeable application for empathy response towards synthetic pain for assistive robots application.

Building on this implementation and critical proof-of-concept work, future research will extend the pain acknowledgement and responses further by integrating sensor data across multiple sensors using more sophisticated data integration. Future works to carefully design experiments that evaluate other kinds of synthetic pain proposed in the paper. Experiments should be designed to accommodate the modelling of robot actions during the visitation of other regions existed in the ASAF framework. The pain activation mechanism will consider recognition of human pain through social cues, such as facial and verbal expressions. Furthermore, measuring the empathy response from human with disabilities would be our primary future study.

## Footnotes

1 The terms mediator and observer which refer to the mediator and observer robots will be used through the rest of the paper.
